# Enhancing the effectiveness of anti‐respiratory virus vaccines by bolstering mucosal immunity and cellular defenses

**DOI:** 10.1002/mco2.616

**Published:** 2024-08-24

**Authors:** Rubing Xue, Sijia Liu, Fangfang Zhou

**Affiliations:** ^1^ The First Affiliated Hospital, the Institutes of Biology and Medical Sciences, Suzhou Medical College Soochow University Suzhou China; ^2^ MOE Laboratory of Biosystems Homeostasis & Protection and Innovation Center for Cell Signaling Network Life Sciences Institute Hangzhou Zhejiang China; ^3^ International Biomed‐X Research Center, Second Affiliated Hospital of Zhejiang University School of Medicine Zhejiang University Hangzhou China

## Abstract

A schematic diagram of intratracheal (IT) boosting, which leads to enhanced mucosal immunity and protective efficacy. IT boosting leads to significant expansion of mucosal neutralizing antibodies, along with robust CD8^+^ and CD4^+^ T‐cell responses. Notably, IT boosting results in substantial and sustained activation of cytokine, natural killer, T, and B‐cell pathways in the lung, contributing to enhanced mucosal immunity and overall protective efficacy.

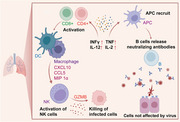

1

Existing SARS‐CoV‐2 vaccines offer reasonable protection against serious illness; however, they are less effective in preventing infections caused by Omicron subvariants.^1^ Intramuscular administration of mRNA and adenovirus vector‐based vaccines often fails to stimulate robust mucosal immunity, prompting the need for innovative strategies to enhance protection and block transmission. A recent study by McMahan et al. addressed the limitations of current SARS‐CoV‐2 vaccines, particularly their minimal protective effects against Omicron subvariants, with a focus on enhancing mucosal immunity.^2^ This research explored a novel vaccination method using IT administration of a bivalent vaccine targeting SARS‐CoV‐2. This method significantly enhances mucosal antibody production and cell‐mediated immunity, offering near‐complete protection in macaques and markedly improving vaccine efficacy.

To assess the robustness of mucosal and peripheral immune responses before and after boosting, 40 adult rhesus macaques were given an initial dose of Ad26.COV2.S vaccine,^3^ followed by a booster dose of either a bivalent Ad26 vector vaccine or a bivalent mRNA vaccine, administered via intramuscular, intranasal, or IT routes. Subsequent to the booster dose, the animals were subjected to a large quantity of the SARS‐CoV‐2 BQ.1.1 strain to evaluate the vaccine's performance against this specific variant. The IT‐administered Ad26 booster offered almost complete protection against viral challenge, showing much better results when compared to other boosting methods. This heightened level of protection was closely linked to robust activation of mucosal antibody and cell‐mediated immunity. Notably, the IT approach led to increased expansion of these immune responses than those achieved with the intramuscular and intranasal routes, offering effective protection against various SARS‐CoV‐2 variants. IT boosting induced stronger mucosal humoral responses, underscoring the critical importance of these responses in vaccine efficacy. The safety of this booster strategy was confirmed by the minimal pathology observed in the vaccinated groups, with no indications of pulmonary fibrosis, further validating its efficacy and safety profile. This approach led to the robust and sustained activation of key immune pathways, including cytokines, natural killer cells, T cells, and B cells, in the lung (Figure 1). This activation was instrumental in enhancing mucosal immunity and the overall protective efficacy of the vaccine, demonstrating the benefits of IT boosting for generating a strong immune response.

**FIGURE 1:**
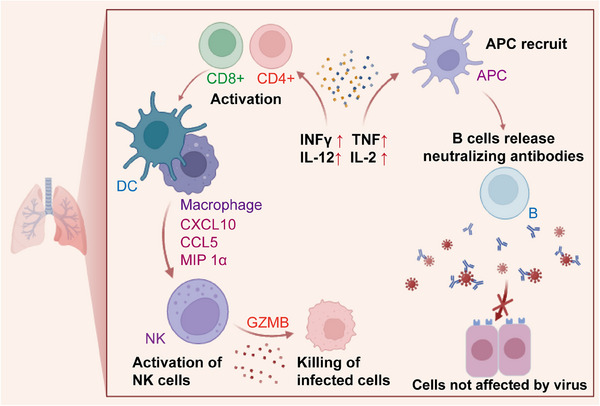
A schematic diagram of intratracheal (IT) boosting, which leads to enhanced mucosal immunity and protective efficacy. IT boosting leads to significant expansion of mucosal neutralizing antibodies, along with robust CD8^+^ and CD4^+^ T‐cell responses. Notably, IT boosting results in substantial and sustained activation of cytokine, natural killer, T, and B‐cell pathways in the lung, contributing to enhanced mucosal immunity and overall protective efficacy.

Another recent study by Uddbäck et al. demonstrated the significant role of CD8^+^ resident memory T cells (TRMs) in reducing viral loads, limiting immunopathology, and preventing viral transmission, unveiling a crucial aspect of immune protection against respiratory viruses.^4^ Although vaccines against respiratory viruses have traditionally focused on inducing antibody responses, this groundbreaking study revealed the pivotal contribution of cellular immunity to both individual‐level and population‐level protection. This study primarily focused on pre‐existing CD8^+^ TRMs and their impact on both host susceptibility and their potential to transmit the virus. This finding challenges the conventional view that CD8^+^ TRMs primarily mitigate viral loads and immunopathology following direct infection. Instead, these findings suggest that these cells can substantially reduce the likelihood of infection and shorten its duration, even without the presence of virus‐specific antibodies.

This study utilized a robust mouse model simulating natural transmission of parainfluenza virus to explore the role of CD8^+^ TRMs in the respiratory tract in establishing herd immunity. The research uncovered that TRM‐mediated protection involves interferon‐γ (IFN‐γ), an antiviral cytokine, and changes in the transcriptional activity of respiratory tract epithelial cells. Importantly, strategically positioned tissue‐resident CD8^+^ TRMs at the site of viral entry and replication have emerged as potent defenders against respiratory viruses, showcasing their ability to not only protect the host from severe disease but also limit viral spread in the population. The durability of TRM‐mediated protection was a key aspect explored in the study.^5^ Different vaccination schedules, encompassing live‐attenuated and replication‐deficient vectors, were assessed for their capacity to stimulate mucosal TRMs. The results demonstrated that TRMs can provide long‐lasting protection, with some immunization strategies maintaining efficacy even after 6 months. Furthermore, the study delved into the role of IFN‐γ, showing its critical importance in limiting the window of respiratory virus transmission. This research highlights that TRM‐mediated protection is more reliant on IFN‐γ‐induced antiviral states rather than direct cytolytic activity, challenging existing theories on the mechanisms underlying CD8^+^ T‐cell effector activity.

Together, these studies represent significant progress in generating comprehensive defense responses against evolving viral variants and contribute to a broader understanding of immunization strategies. While these studies have allowed for significant advancements in the development of vaccines against respiratory viruses, particularly SARS‐CoV‐2, there are several limitations and potential avenues for future research. First, the translational potential of IT boosting with Ad26‐based vaccines from macaque models requires thorough investigation to ensure its safety and efficacy in humans. Additionally, the long‐term durability of induced mucosal and cellular immune responses, especially in the context of emerging variants, remains uncertain and requires longitudinal studies in both animal models and clinical trials. Moreover, understanding the interplay between mucosal immunity, cellular defense, and systemic immune responses could provide deeper insights into the mechanisms underlying vaccine‐induced protection and guide the development of future vaccines. Exploring innovative delivery methods, adjuvants, and vaccine platforms tailored to mucosal immunity and cellular responses could offer promising strategies for overcoming current limitations and achieving broader and more durable protection against respiratory viruses. Furthermore, investigating the potential synergistic effects of combining mucosal and cell‐mediated vaccine approaches may offer a comprehensive strategy for eliciting robust and long‐lasting immunity, thereby addressing the evolving challenges posed by respiratory virus pandemics.

## AUTHOR CONTRIBUTIONS

Rubing Xue conceived and drafted the manuscript and drew the figure. Sijia Liu and Fangfang Zhou discussed and revised the manuscript. All authors have read and approved the article.

## CONFLICT OF INTEREST STATEMENT

The authors declare they have no conflicts of interest.

## Data Availability

Not Applicable
